# The Performance of a Passive Autoranging Method for a Photonic Current Transducer

**DOI:** 10.3390/s24103183

**Published:** 2024-05-17

**Authors:** Grzegorz Fusiek, Burhan Mir, Pawel Niewczas

**Affiliations:** Department of Electronic and Electrical Engineering, University of Strathclyde, Glasgow G1 1XQ, UK; burhan.mir@strath.ac.uk (B.M.); p.niewczas@strath.ac.uk (P.N.)

**Keywords:** fiber Bragg grating, piezoelectric transducer, photonic current transducer, current transformer, autoranging, extended dynamic range

## Abstract

This paper reports on the testing and evaluation of a passive autoranging (AR) method designed to dynamically extend the measurement range of a photonic current transducer (PCT) to pave the way toward a realization of a combined metering- and protection-class current sensor. The PCT utilizes a current transformer (CT), a piezoelectric transducer (PZT), and a fiber Bragg grating (FBG) to enable current measurement at multiple points in an electrical power network whereby multiple sensors are deployed and interrogated serially using a single optical fiber. The autoranging technique relies on incorporating static MOSFET switches to instantaneously short individual serially connected CT burdens in response to a measured current magnitude exceeding pre-set thresholds. The AR circuit switching events produce distinctive signal features that are used by the proposed switching algorithm to apply appropriate scaling factors to reconstruct the measured current from the optical signal. It is shown through laboratory experiments that the AR circuit correctly reacts to pre-set burden current thresholds of 130% of the nominal value and 22 times the nominal value, signifying its “metering” and “protection” range boundaries. The circuit reaction time is below 4 ms, rendering it suitable for standard power system protection purposes. Moreover, the operation of the AR circuit is demonstrated for burden currents of up to 100 A for over 1 s, satisfying a test procedure for the secondary CT circuit, as required by some power system operators. It is demonstrated that the proposed switching algorithm allows for a correct reconstruction of the burden currents from the optical signal acquired by the FBG interrogator, offering the potential to realize a dual-class optical current sensor.

## 1. Introduction

Accurate measurements of voltage and current in electrical power networks are essential for metering and protection purposes, as defined by IEEE and IEC standards. Conventional iron-core voltage and current transformers (VTs and CTs) prevail as the primary technology in the industry for voltage and current measurement across transmission and distribution networks. However, they present notable drawbacks, including their considerable size and weight, which impact substation dimensions and installation cost. Moreover, they rely on secondary copper leads for connection to substation equipment which lack galvanic isolation and pose safety risks, such as the danger of explosion due to the oil-filled insulation systems in use [1,2]. Consequently, network operators are exploring alternative solutions, such as optical sensors, to address these shortcomings.

In parallel, advancements in optical sensing technologies have witnessed the emergence of fiber-optic current sensors (FOCSs) based non-conventional instrument transformers (NCITs) which are unique for their reduced weight, environmental safety, and increased bandwidth [3,4]. However, their deployment has been limited by vulnerabilities to environmental conditions, their high cost, and, in some cases, a need for external power supplies, constraining their widespread applicability [5]. Conversely, FBG/PZT-based optical current sensors have garnered attention due to their compact size and reduced weight, inherent electrical isolation, immunity to electromagnetic interference, and serial multiplexing capability [6,7,8,9,10]. FBG/PZT-based optical current sensors can be serially multiplexed to combine multiple FBGs into a single optical fiber, simplifying the monitoring of a large network and saving on installation costs. A single interrogator located centrally in a substation can be used to offer instantaneous, synchronized current data from a wide-area power network, enabling innovative power network control or protection features [6].

However, FBG/PZT-based optical current sensors (OCSs) and photonic current transducers (PCTs) face constraints in dynamic range and accuracy when compared to fiber-optic current sensor (FOCS)-based NCITs [5,11,12]. Notably, meeting the combined 0,2S and 5P20 class standards [13], which demand both a wide dynamic range and high accuracy across an extended current range, remains a challenge for current technology [14,15]. The PCT technology solution previously proposed by the authors adheres to the IEC/IEEE standards, demonstrating the potential to comply with either the 5P protection class or the 0,2 metering class. However, it currently lacks the capability to meet the requirements of the 0,2S class or concurrently fulfill the requirements of both the 0,2 and 5P classes within a single device.

One proposed solution for extending the current measurement range of the PCTs involves replicating the measurement chain, resulting in separate PCTs for metering and protection classes. However, this approach increases bulk, weight, size, and cost due to the use of two or more sensors. Furthermore, employing two or more FBGs reduces the overall optical bandwidth, limiting the number of sensors that can be addressed by a single interrogation unit. The novel autoranging technique proposed by the authors of [15] has the potential to concurrently achieve both 0,2S and 5P20 classes with a single photonic current transducer (PCT) by dynamically extending the measurement range.

The foundational work by Mir, Niewczas, and Fusiek [15] introduced a novel technique for autoranging an FBG/PZT-based optical current sensor in which a passive autoranging (AR) circuit can be integrated with a CT, burden resistors, and a low-voltage transducer (LVT), resulting in an extended-range PCT. The LVT is a low-voltage variant of a PZT/FBG transducer [9,10,15]. The integration of autoranging capabilities into these sensors presents a breakthrough, enabling passive adjustments to measurement sensitivity and range. The dynamic range of the PCT is normally limited by the LVT’s maximum withstand voltage and the interrogator’s noise floor. Presently, the sensor construction limits the LVT’s maximum voltage capability to approximately 20 V (RMS). The autoranging enhancement brings the potential to position the FBG/PZT-based optical sensor as a competitive alternative to FOCS-based NCITs, additionally offering multiplexing and remote interrogation capabilities. To validate this approach and demonstrate circuit characteristics, a prototype AR circuit was previously implemented in a laboratory using two MOSFET switches and serially connected burden resistors [15].

This methodology showcases the feasibility of extending measurement ranges and enhancing sensor sensitivity while also ensuring the protection of the burden resistors against 100 A test currents. This paper investigates the performance of the proposed autoranging technique. As before, the PCT utilizes a CT, PZT, and FBG. The autoranging technique incorporates static MOSFET switches to instantaneously short individual serially connected CT burdens (1 Ω and 16 Ω in the modified circuit) [15]. The circuit is now complemented by an algorithm capable of detecting distinct switching events produced by the AR circuit to apply appropriate scaling factors to reconstruct the measured current from optical signals. It is shown through laboratory experiments that the AR circuit correctly reacts to a burden current threshold of 130% of the nominal current value and at a current 22 times the nominal value with a reaction time below 4 ms. The operation of the AR circuit is also demonstrated for secondary test currents of up to 100 A over a duration of more than 1 s. It is demonstrated that the proposed switching algorithm allows for the correct reconstruction of burden currents from signals acquired by an FBG interrogator, offering the potential to realize a dual-class optical current sensor.

## 2. Materials and Methods

As highlighted in the Introduction, the autoranging technique, which was previously proposed by the authors in [15], involves multiple CT burden resistors that are connected in series. These resistors are associated with static MOSFET switches that allow for their immediate shorting when pre-set thresholds are breached and their un-shorting when the secondary current reduces. Control of the MOSFETs is achieved passively through a modular, low-power comparator circuit that draws its energy from the CT, powering up within a short fraction of the 50 Hz cycle. Changes in the PCT’s sensitivity (or gain) are then instantly detected by the central FBG interrogator, which analyzes the optical signals in real time. This information is used to apply appropriate scaling factors, enabling the usage of a single CT to cover a wide dynamic range of measurements, potentially enabling a combined metering- and protection-class current sensor.

**Figure 1:**
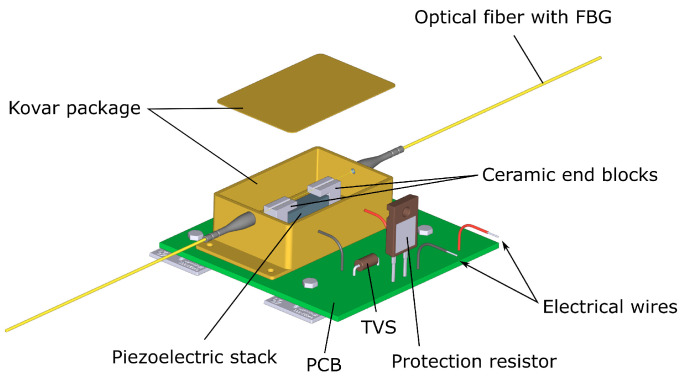
A conceptual drawing of a low-voltage transducer (LVT) with overvoltage protection circuit components mounted on a PCB.

**Figure 2:**
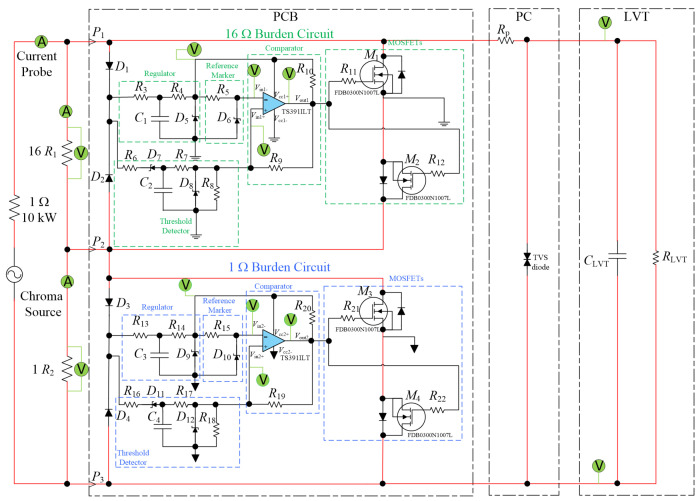
A circuit diagram of the experimental setup including the Chroma voltage source, the Chroma load, the burdens, the autoranging circuit PCBs, the LVT, and its protection circuit (PC).

The proposed PCT device comprises a dual-class CT with two burden resistors, an AR circuit, a low voltage transducer (LVT), and an overvoltage protection circuit (PC). The LVT overvoltage protection circuit contains a protection resistor, (R_p_) and a transient-voltage-suppression (TVS) diode that are connected across the LVT (see Figure 1 and Figure 2). The LVT is built as a separate, hermetically sealed unit, as shown in Figure 1. It comprises a low-voltage piezoelectric multilayer stack and a bonded fiber Bragg grating (FBG) sensor [9,15].

The FBG within the metallic LVT package is suspended between two ceramic arms which are attached to a PICMA^®^ stack (5 × 5 × 18 mm) from Physik Instrumente (PI) (Karlsruhe, Germany) [16]. The operating voltage range of the PICMA^®^ stack is from −30 V to 120 V. A resonant frequency of 70 kHz can be assumed for an unclamped and unloaded component. The stack can reach its full displacement in approximately 4.8 μs after a driving voltage change [16]. The LVT’s construction ensures strain amplification, while its operating voltage range is limited to ±30 V through the protection circuitry to avoid piezoelectric stack depolarization and permanent damage [9,15]. The strain proportional to the input voltage is generated in the stack and imparted on the FBG, causing shifts in its peak wavelength. The instantaneous peak wavelength can be calibrated in terms of the measured voltage, while from the average wavelength, the local sensor temperature can be derived and used for temperature compensation of the sensor voltage readings [9,15].

## 3. Results

### 3.1. Performance Requirements

The proposed system is designed for CTs with a 1 A-rated secondary current. The goal is to satisfy the requirements of the combined metering (0,2S) and protection 5P20 classes within one PCT device. The circuit aims to achieve a reaction time to the input current due to a power system fault of within 4 ms. The CT used for metering needs to be sufficiently accurate within a range of 1–120% of the rated current, while for protection purposes, the CT should be sufficiently accurate at the rated current and at the rated accuracy limit factor. The CT should also withstand thermal current tests for 1 s.

Consequently, the proposed autoranging circuit consists of two burden resistors with dedicated MOSFET switches and control circuits. In a proof-of-concept technology demonstrator, 1 Ω and 16 Ω burden resistors were used and connected in series with the 1 A nominal current through the burdens. The unit is required to maintain its metering accuracy up to 120% of the nominal current, i.e., 1.2 A. For the 5P20 protection class, the unit is required to measure currents up to a rated accuracy limit of 20 A on the secondary CT winding.The design requirement is such that the MOSFET switch connected to the 16 Ω burden resistor should activate (close) when the input current crosses the low current range. In the present case, the low current range is assumed to be 1.3 A, which is around 10% greater than 1.2 A. This corresponds to 20.8 V across the 16 Ω burden resistor. Meanwhile, the MOSFET switch connected to the 1 Ω burden should be inactive (open) at this point. (Note that the combined voltage across the LVT just before the range is switched is 20.8 V + 1.3 V = 22.1 V). When the input current exceeds the 22 A threshold (10% greater than the required 20 A rated accuracy limit), the first MOSFET switch should close to protect the 1 Ω burden resistor from overheating. Note that 22.1 V (RMS) is equivalent to 31.25 V (peak), and so the selection of the TVS diode (if it is desired to be used as a second line of defense for the LVT) should be such that the clamping voltage, including any tolerances, should be above that value.

Assuming a rated current of 1 A, the voltage across both burdens will be 17 V (the voltage seen by the LVT). At 120% of the rated current (1.2 A), the voltage across the burden resistors will be around 20.4 V. A current of 1.3 A will result in a voltage of 20.8 V across the 16 Ω resistor and 1.3 V across the 1 Ω resistor, with a total of 22.1 V (31.25 V peak) measured by the LVT. At this current threshold, the circuit is required to extend the measurement range by shorting the 16 Ω resistor. Consequently, the combined voltage measured by the LVT will be reduced to 1.3 V. This will allow for the current to rise further up to 22 A before the second set of MOSFET switches activates and both burdens are bypassed.

Consequently, if both sets of MOSFET switches are inactive, the device will cover the current range for metering purposes and the rated current for protection purposes (metering (M) range). With the first switch active across the 16 Ω burden, measurements up to the accuracy limit (5P20) for protection purposes will be covered (protection (P) range). When both sets of switches are active, the burden resistors will be protected from overheating during the 100 A thermal current tests (thermal test (TT)).

A summary of the autoranging system requirements is provided in Table 1.

### 3.2. Experimental Setup

**Figure 3:**
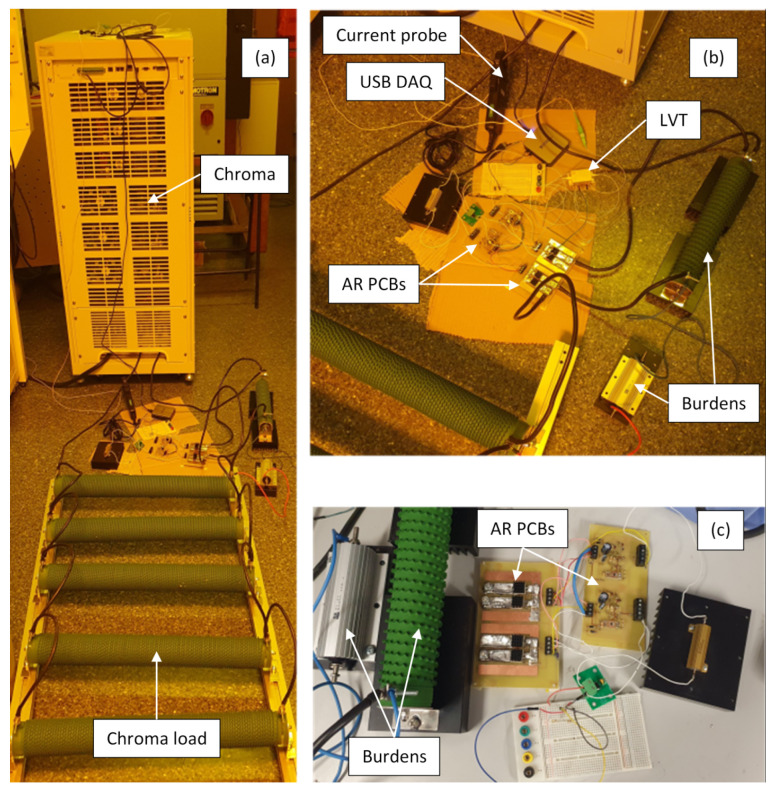
Photographs of the experimental setup: the Chroma source connected to the load, the burdens, and the autoranging circuit PCBs (**a**); a close-up of the burdens, the autoranging circuit PCBs, and the LVT (**b**,**c**).

To experimentally evaluate the AR circuit with the proposed switching algorithm, the individual 16 Ω and 1 Ω burden resistors were equipped with dedicated MOSFET switches and control circuits, as shown in Figure 2. The LVT was connected across the combined burden resistors and was protected against overvoltage using a protection circuit (PC) composed of a 10 Ω protection resistor (R_p_) and a TVS diode. A Chroma 61512 unit (Chroma Systems Solutions, Inc.) was used as a power source. This was connected via a 1 Ω current limiting resistor. Note that the Chroma unit is a voltage source rather than a current source; this poses certain experimental limitations which will be discussed later. Photographs of the experimental setup are presented in Figure 3.

The current in the burden resistors was measured and monitored using a clamp-on TA167 current probe (Pico Technology) with an accuracy of ±1% for the used measurement range [17]. The output of the current probe and the voltage across the LVT were captured using an NI USB-6003 DAQ at 4 ksps to match the LVT interrogation speed (4 kHz). Because the DAQ input voltage range is ±10 V, the LVT voltage was scaled down using an additional voltage divider formed using 100 kΩ and 1 kΩ resistors. The divider will not be required when the LVT is used with an AR circuit and a relevant dual-class CT in real applications.

### 3.3. Initial Tests

Initial tests of the AR circuit’s operation were carried out with currents ranging from 1 A to 100 A which were injected into the burden resistors from the Chroma source; this involved verifying the correct activation of the switches depending on the magnitude of the measured current.

**Figure 4:**
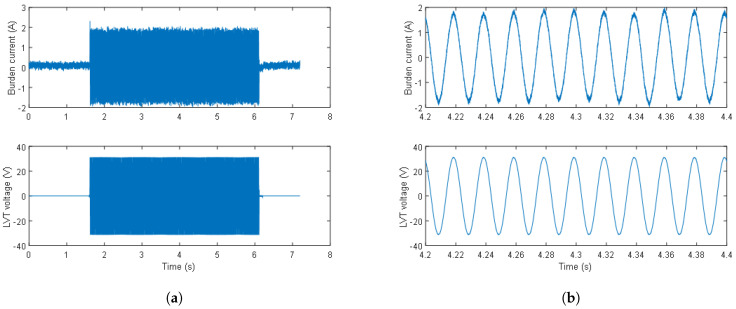
Burden current and LVT voltage waveforms at 1.3 A just before the activation of the first switch (**a**), and a zoom of the burden current and LVT voltage waveforms at 1.3 A just before the activation of the first switch (**b**).

An example of the 1.3 A current flowing through the burdens (boundary condition) and the resultant LVT voltage are shown in Figure 4a. The LVT voltage is 22.1 V (31.2 V peak). The waveforms show no activation of the first switch (across the 16 Ω burden), demonstrating the correct operation of the AR circuit for currents up to 130% of the nominal current (1 A). Waveforms of the same signals zoomed across the time axis are shown in Figure 4b. The apparent high-frequency noise and sub-50-Hz modulation on the current waveform were caused by a pickup from the current probe and its wire leads. This was due to the high current range of the available current probe whereby a current of 1.3 A constitutes only 0.65% of the current probe range. This interference is not visible on the LVT voltage waveform as the voltage across the sensor is nearly 100% of the sensor measurement range in this case.

**Figure 5:**
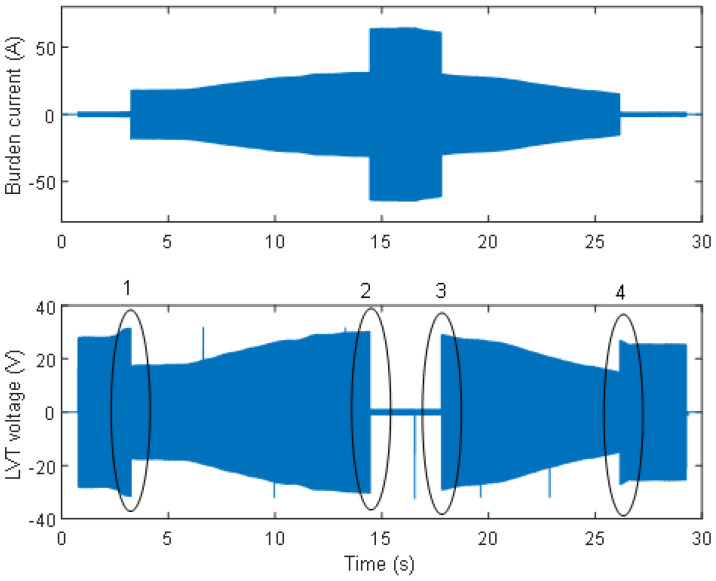
Burden current and LVT voltage waveforms at currents ranging from 1 A to 50 A, showing the activation of both switches. Instances 1 and 4 are switching on and off of the first switch; instances 2 and 3 are switching on and off of the second switch.

To demonstrate the operation of the AR circuit switching during a current amplitude sweep up to 50 A, another experiment was conducted, as described below. The results of this investigation are presented in Figure 5, showing the activation and deactivation of both switches. At the 18 Ω load, the voltage generated from the Chroma unit was first set to achieve a current of 1.1 A through the burden resistors, which resulted in an LVT voltage of 18.7 V. The output voltage from the Chroma unit was then adjusted manually to continuously increase the current in the circuit rather than provide a step change. The current was increased from its initial value until the first switch was activated after it crossed a threshold of approximately 1.3 A. At that time, the voltage across the LVT was reduced from 22.2 V to 11.7 V due to a change in the Chroma load from 18 Ω to 2 Ω when the 16 Ω resistor was shorted. Note that this is the limitation of the experimental setup when using the Chroma source, which is a voltage source rather than current source. In a real application, when using a CT (a current source), the voltage across the LVT should drop to around 1.3 V (1 Ω × 1.3 A). Because the voltage set on the Chroma unit at the time of switching is the same as before the switch activation, the current value is nine times larger after switching than it would be in a real application. For this reason, further ramping up of the voltage can only be observed above 11.7 V across the LVT, as can be seen in Figure 5 (Instance 1).

After crossing the 11.7 V across LVT, the voltage on the Chroma unit was ramped up again until the next threshold at which a current of approximately 22 A through the 1 Ω burden was achieved. At this point, the second switch was activated (Instance 2 in Figure 5), and the voltage across the LVT dropped to approximately 0.7 V due to the change in the Chroma load from 2 Ω to 1 Ω. As described previously, the voltage set on the Chroma unit at the time of switching was the same as before the second switch activation, and the current increased from 22 A to 44 A after switching. Since 4 MOSFETs with a resistance of 4 mΩ per MOSFET are conducting when both switches are activated, the voltage across the LVT is equal to 0.704 V (4 × 4 mΩ × 44 A). This agrees with the waveforms shown in Figure 5. The switching time was below 0.5 ms in this case. Next, the voltage on the Chroma unit was gradually decreased so that the current flowing through the burdens reached a level of approximately 43 A, switching off the second switch (Instance 3 in Figure 5). At that time, the Chroma load was increased from 1 Ω to 2 Ω, and the voltage across the LVT was increased to approximately 21 V. Further decreases in the Chroma voltage allowed the first switch to deactivate (Instance 4 in Figure 5) at 10.8 A flowing through the 1 Ω burden and changing the Chroma load from 2 Ω to 18 Ω, which resulted in the increase of the LVT voltage to approximately 20.4 V. The voltage was then lowered to near the nominal value.

As can be seen in Figure 5, the AR intermittent circuit switching occurred every 3–4 s. This is due to the discharging of a main capacitor that holds a charge for the control circuit to operate. It should be noted, however, that in real applications, a fault would clear well before the 3–4 s time, so this is not an issue for a real-world PCT application. However, as mentioned earlier, the reaction time of the circuit can be adjusted if the switches are to be active for less than 3–4 s. Short-time voltage impulses generated by the switching of the comparator and the transistors are below 100 ms in duration, and they are not considered to generate any significant power to be dissipated in the burden resistors [18]. Provisionally, they are limited to the LVT safe voltage range of approximately ±30 V peak by means of a protection resistor and TVS diode to protect the LVT from depolarization and damage due to overvoltage conditions. It should also be noted that rapid changes in the burden signals, such as those produced by the Chroma source, are unlikely to occur in real applications. It is envisaged that a dedicated CT will smooth and filter the input current and changes in the amplitude of the secondary CT current will not be as rapid as those generated by Chroma. Therefore, the operation of the AR circuit when monitoring the output of a dedicated CT will additionally be investigated as soon as the CT becomes available.

**Figure 6:**
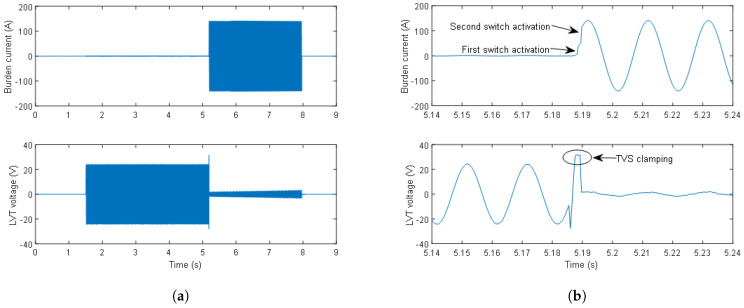
Burden current and LVT voltage waveforms at 100 A after activation of both switches: (**a**) and a zoom of burden current and LVT voltage waveforms at 100 A after activation of both switches (**b**).

The next experiment involved testing the AR circuit’s capability to respond to a sudden change in the burden current magnitude from nominal conditions to 100 A. An example of the AR circuit’s reaction to the 100 A burden current is shown in Figure 6. The initial current is set to a nominal value of 1 A, followed by an injection of 100 A from the Chroma unit. The LVT voltage drops from the nominal 17 V to 0.9 V within 3 ms, as can be seen in Figure 6a,b. The sequential operation of the switches can be clearly seen in Figure 6b. The first switch activation is after 0.6 ms, while the second switch is activated 1 ms after the first switch. The switching time for both switches is 0.25 ms, resulting in a total delay time of 2.2 ms.

In Figure 6a, when both switches are on, a small increase in the amplitude of the LVT voltage with time can be observed. This effect was caused by the heating of the conducting MOSFETs due to insufficient cooling with the heatsinks that are currently used, and the fact that the resistance of the conducting MOSFETs increases slightly with temperature. The effect is minimal within the required first second of operation and can be further improved with the application of larger heatsinks. The LVT protection circuit operation is correct, as can be seen in Figure 6b, where the TVS clamping is highlighted.

## 4. The Conceptual Design of the Switching Algorithm

### 4.1. The General Requirements of the Switching Algorithm

The experiments carried out as described in the previous section revealed that the operation of the AR circuitry is as expected, and each switch activation or deactivation results in rapid changes in the current through the burden resistors and the voltage across the LVT to signals with different magnitudes. These sudden signal changes due to the MOSFETs switching are deemed to be distinct from network faults in real applications. MOSFET switches are capable of rapid switching in the sub-millisecond region. Therefore, any voltage changes at the LVT due to MOSFET actuation can be distinguished from changes in the signal magnitude caused by network events, such as those encountered during fault conditions. Fault conditions on the network occur at least an order (or even up to three orders) of magnitude slower than surges caused by MOSFET switching as they are additionally filtered by the CT acting as a low-pass filter [15]. Since AR circuitry switching will always limit the amplitudes of the LVT voltage to ±30 V for all measurements including metering and protection ranges, an appropriate algorithm allowing one to distinguish the individual AR switch activation to apply a correct current measurement scale is required.

To fully utilize the autoranging system, the LVT interrogator requires a high-speed voltage sensing capability, with its firmware being able to identify a sudden change in voltage, ΔV/Δt, at the LVT. Advanced signal processing techniques can be deployed to accurately measure the rate of voltage change over time to detect the actuation of the MOSFET switches through the resulting voltage change at the burden. For example, if a network fault current occurs and the current flowing through the CT exceeds the threshold level, the MOSFET switches will actuate, and the interrogator will detect the sudden voltage drop at the burden, indicating the need for a change in measurement scale [15].

### 4.2. The Initial Design of the Switching Algorithm

In the prototype autoranging system, the LVT is interrogated using an I-MON 256 USB unit with an interrogation speed of 4 kHz, resulting in a time resolution of 0.25 ms. At this interrogation speed, some very narrow impulses generated during AR circuit switching may not be visible on the optical signal. They will also be flattened and limited by the TVS diode to remain within the LVT safe voltage level (±30 V). The maximum reaction time to a fault current for the AR system needs to be within 4 ms. This means that the detection of the AR circuit switching moment needs to be based on monitoring the rate of change rather than the RMS or amplitude values of the LVT signal. It should also be noted that although there will be access to the RMS or historical amplitude data (prior to the switching), the reaction time requirement of 4 ms provides a maximum of 16 samples of 50 Hz signals when sampled at 4 kHz. This is equivalent to one-fifth of the sine period in this case, which is not sufficient for predicting the RMS or amplitude values occurring just after the switching event. Although there are various methods for the fast estimation of RMS values, they require data from at least a quarter of a sine wave period [19]. For this reason, in the initial design of the switching algorithm, it is proposed that the first derivative of the LVT signal will be monitored, which should provide distinctive spikes at each switch activation instance. The spikes, when crossing a set threshold, would generate a logical “1” or “True” impulse that could be further processed by the algorithm. From the moment of the first switch activation, the algorithm should apply an appropriate measurement scale to account for the change in the amplitude of the measured voltage across the burdens. The relevant scale factor would have to be applied until the switch is deactivated after 3–4 s. After this instance, the algorithm should return to the previous measurement scale factor.

**Figure 7:**
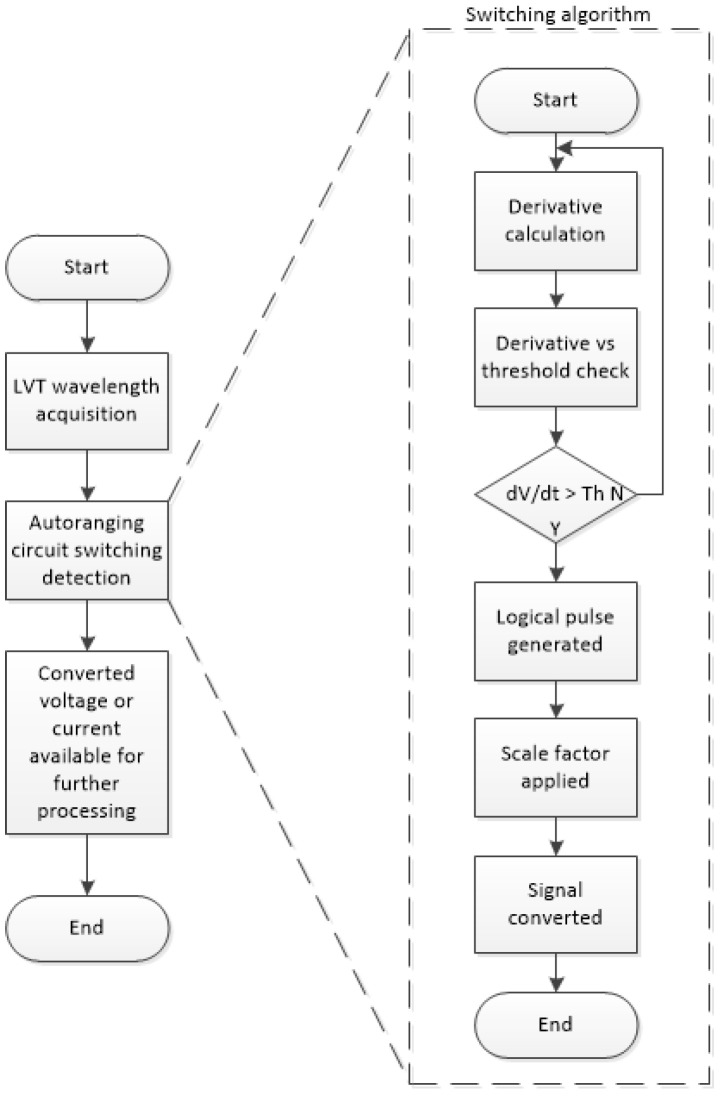
Functional diagrams of LVT signal processing, including autoranging circuit switching detection.

Functional diagrams of the switching algorithm for the autoranging circuit and LVT operation are presented in Figure 7.

Consequently, the proposed algorithm’s functional blocks were implemented in LabVIEW 2023 software and tested with the electrical and optical signals acquired from the AR system.

### 4.3. Testing the Switching Algorithm with the LVT Connected

To test the proposed switching algorithm, it was implemented in LabVIEW software for an offline analysis of the signals logged during the AR circuit tests when the LVT was connected to the AR circuit. The DAQ and LVT signals were logged at 4 kHz. Due to the physical spread of the experimental setup, the acquisition of optical and electrical signals was not hardware-synchronized during the experiments. However, the same technique of signal differentiation applied to the electrical signals was applied to the LVT optical signals. For the optical signals, the electrical signal scaling factors were updated with those taking into account the wavelength-to-voltage conversion, assuming an LVT sensitivity to voltage of 14 pm/V.

**Figure 8:**
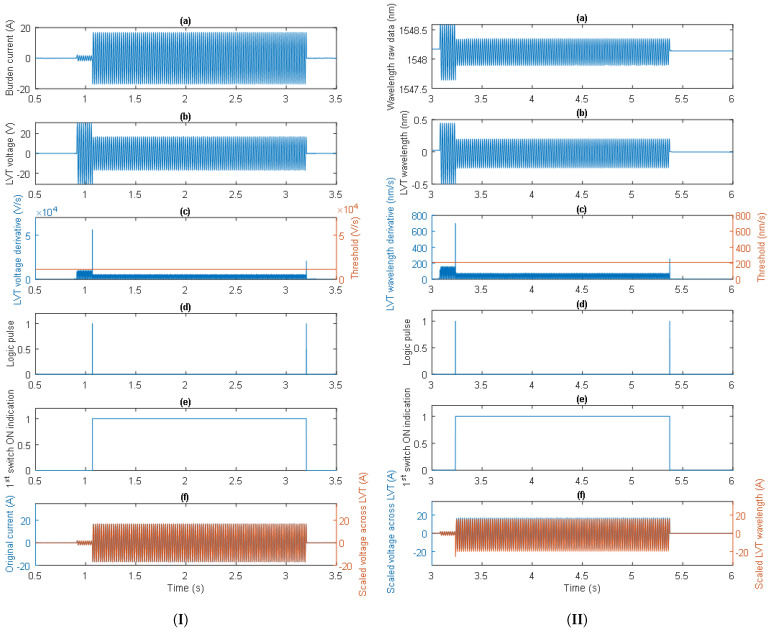
The proposed switching algorithm processing the electrical signals (**I**) and the optical signals (**II**).

The operation of the proposed algorithm is shown in the examples given in Figure 8, where the consecutively processed signals are depicted. The signals in Figure 8I(a,b) show the burden current and the LVT voltage, respectively. The burden current is initially equal to 1.2 A, while the LVT voltage is 20.4 V. The current is then rapidly increased to cross the first switch activation threshold, resulting in a rise in the burden current to 12.1 A and a drop in the LVT voltage to 12.1 V. The LVT voltage derivative generated a high-amplitude spike at that time which was above the set threshold, as shown in Figure 8I(c). The threshold value was set to 20% of the maximum value of the LVT voltage derivative. This generated a logical 1 pulse in the software, as shown in Figure 8I(d), indicating the state of the first switch as “ON” (logical 1 in Figure 8I(e)). During this time, the LVT voltage was multiplied by a scaling factor of 1 as the 16 Ω burden was shorted out. This state lasted for approximately 2 s, after which time the burden current was switched off, and the switch was deactivated. A comparison of the original burden current and the scaled LVT voltage expressed in amps is shown in Figure 8I(f).

Similarly, the optical LVT signals can be processed as shown in Figure 8II. The signals in Figure 8II(a,b) show the LVT raw wavelength and the LVT wavelength with the DC offset removed, respectively. At a burden current initially equal to 1.2 A and an LVT voltage of 20.4 V, the LVT wavelength is approximately 0.31 nm. The current is then rapidly increased to cross the first switch activation threshold, resulting in an increase in the burden current to 12.1 A and a drop of the LVT voltage to 12.1 V. The LVT wavelength derivative generated a high-amplitude spike at that time, which was above the set threshold, as shown in Figure 8II(c). The threshold value was set to 30% of the maximum value of the LVT wavelength derivative. This generated a logical 1 pulse in the software, as shown in Figure 8II(d), indicating the state of the first switch was “ON” (logical 1 in Figure 8II(e)). During this time, the LVT wavelength was multiplied by a scaling factor of 1 as the 16 Ω burden was shorted out and by the LVT wavelength-to-voltage sensitivity factor (14 pm/V). This state lasted for approximately 2 s, after which time the burden current was switched off and the switch was deactivated. A comparison of the scaled LVT wavelength and the scaled LVT voltage, both expressed in amps, is shown in Figure 8II(f). As can be seen, the optical signal has some non-symmetry in comparison to the LVT voltage, which might be caused by the aging/de-aging effect in the LVT [20,21,22]. Further investigation of these effects is required.

**Figure 9:**
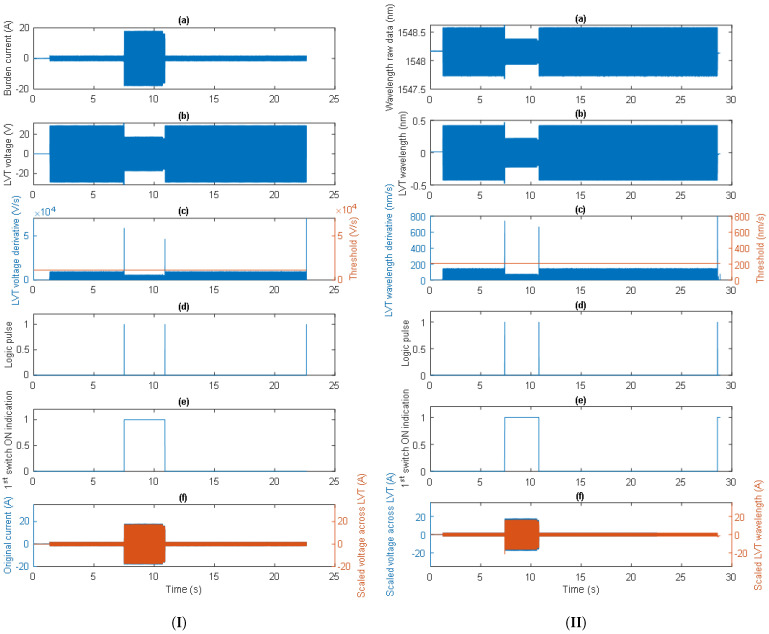
The proposed switching algorithm processing electrical signals (**I**) and optical signals (**II**).

The capability of the algorithm in switching signals is demonstrated in another example shown in Figure 9 fro when the experiments were repeated for similar burden currents. Clearly, this time as well, the operation of the AR system was correct, and the current waveform was properly reconstructed from the optical signal.

**Figure 10:**
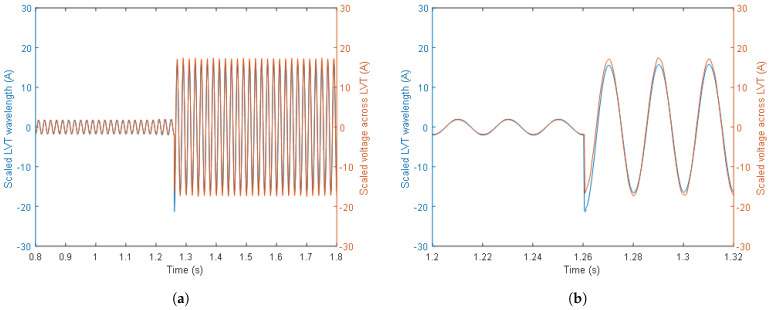
A comparison of the scaled LVT wavelength and the scaled LVT voltage during the first switch activation (**a**) and a greater close-up (**b**).

A close-up of the comparison between the scaled LVT wavelength and the scaled LVT voltage presented in Figure 9II(f) during the first switch activation is shown in Figure 10.

It can be seen from Figure 10 that the electrical signals are relatively well replicated by the optical signals. However, the optical signal has some slight non-symmetry in comparison to the LVT voltage which might be caused by the aging/de-aging effects in the LVT. There is also some slight inaccuracy in the conversion of the LVT wavelength and voltage magnitudes into the burden current. It should be noted, however, that an LVT wavelength-to-voltage sensitivity of 14 pm/V was assumed in the presented analysis. Since the LVT sensitivity is slightly different for each LVT unit and varies with temperature, it is envisaged that a proper calibration of the LVT would produce more accurate results and smaller measurement errors.

## 5. Discussion

Examples of the correct operation of the proposed AR circuit and the switching algorithm were shown in the previous sections. However, it is possible to identify some “special cases” in which the proposed algorithm might struggle to detect AR circuit switching events based only on the rate of change in the optical signal.

One problematic situation occurs when the switching of the AR circuit, especially of the first switch, falls exactly at the zero-crossing point of the current/voltage signals. Even though the switch may operate correctly and the current and voltage signals can be scaled at the LVT as expected, the algorithm, in its most basic form, is incapable of recognizing the switching moment based on the signal derivative alone as such changes do not produce a distinctive change in the signals and therefore are not detectable by the derivative. Consequently, a pulse indicating such a change is not generated. In such a case, the algorithm will continue to use incorrect scaling factors for calculating the measured current, which may result in a false indication of the fault condition.

Yet another problematic case might occur when the LVT voltage is at the same level before and after the activation of the first switch. The discussed switching detection problem may be especially pronounced if the following conditions were to occur simultaneously: the LVT voltage is close to the switching threshold (boundary condition); the fault event produces the same voltage after the first switch activation; and the switching moment falls exactly at the peak of the LVT voltage or in the LVT voltage waveform region flattened by the TVS diode. Further investigation into similar cases needs to be conducted.

In addition to the above cases, there might be situations in which the fault is not cleared within the indicated time, and there can be multiple pulses generated due to the intermittent switching of the AR circuit. In such a case, it may happen that the switching will be falsely recognized by the algorithm, resulting in the usage of an incorrect scaling factor for the calculation of the measured current, which will translate into a false recognition of the fault condition. Similarly, erroneous switching detection can take place when multiple impulses are produced by the LVT signal derivative upon the first switch activation.

To prevent this type of malfunction, the switching algorithm cannot rely only on the derivative of the LVT signal but would need to consider, e.g., the historical data to observe the step change in either the amplitude value of the voltage signal or the voltage signal envelope. Further investigation into the switching algorithm operation and its improvement to properly function at the aforementioned conditions is required. Further work is required to quantify the probability of such occurrences to assess their impact on operation and to propose a backup algorithm to counter this problem or reset the system to its correct state.

## 6. Conclusions

In this paper, an investigation of the performance of a passive autoranging circuit aiming to extend the dynamic measurement range of a photonic current transducer to realize a combined metering- and protection-class current sensor was presented. Two-stage circuitry incorporating two burden resistors, 1 Ω and 16 Ω, was used to demonstrate the AR circuit’s performance when connected to an LVT. It was shown that the circuit correctly reacts to measured burden current threshold breaches of 130% of the nominal value and at a current of 22 times the nominal current with a reaction time below 4 ms. The correct operation of the AR circuit was also demonstrated for a thermal current of up to 100 A flowing through the circuit for at least 1 s. It was shown that the proposed switching algorithm allowed for a correct reconstruction of the burden currents from the optical signals acquired by the interrogator, offering the potential to realize a dual-class optical current sensor.

Several special cases were identified that may result in the incorrect operation of the current embodiment of the switching algorithm, resulting in an incorrect calculation of the measured current or a false recognition of the fault condition. Although such events are considered rare, further work is required to quantify the probability of such occurrences to assess their impact on the system’s live operation and to propose a backup algorithm to counter this problem or reset the system to its correct state.

Future work will focus on the investigation of the AR circuit and the performance of the switching algorithm when special cases are present in the measured current waveforms. Afterward, sensor system accuracy tests will be performed.

## Figures and Tables

**Table 1:** Specifications of the autoranging system.

LVT Specifications	
Rated voltage (V)	17
Maximum voltage (Vpk)	32
Sensitivity to voltage (pm/V)	14
Capacitance (µF)	1.5
Resistance (MΩ)	200
Resonant frequency (kHz)	70
**CT secondary current specifications**	
Rated current (A)	1
Accuracy limit factor (ALF)	20
Maximum thermal current (A)	100
Maximum thermal current duration (s)	1
**Aimed accuracy class**	
Protection	5P20
Metering	0,2
Special application	0,2S
**Autoranging circuit specifications**	
Number of switching stages and burdens	2
Maximum reaction time to fault current (ms)	4
Minimum switched ON state duration (s)	1

## Data Availability

All data underpinning this publication are openly available from the University of Strathclyde KnowledgeBase at https://doi.org/10.15129/e9a4ac0e-8e25-4ab6-8b0a-5de63e5e0bdc (accessed on 10 May 2024).
